# Spinal myeloid sarcoma as a first sign of acute myeloid leukaemia: A case report

**DOI:** 10.1016/j.radcr.2025.11.040

**Published:** 2025-12-04

**Authors:** Thomas Saliba, Laurence DeLeval, Sabine Blum, Gerasimos Tsilimidos, Nicolas Danthez, Vincent Dunet

**Affiliations:** a Radiology department, Lausanne University Hospital and University of Lausanne, Lausanne, Switzerland; b Institute of Pathology, Lausanne University Hospital and University of Lausanne, Lausanne, Switzerland; c Oncology department, Lausanne University Hospital and University of Lausanne, Lausanne, Switzerland; d Radio-oncology department, Lausanne University Hospital and University of Lausanne, Lausanne, Switzerland

**Keywords:** Chloroma, Acute myeloid leukaemia, Myeloid sarcoma, Cancer, Spine

## Abstract

Myeloid sarcomas, also called granulocytic sarcomas or chloromas, are rare extramedullary tumours of immature myeloid cells, most often associated with acute myeloid leukaemia (AML). Spinal involvement is particularly uncommon and typically manifests with neurological symptoms due to nerve compression. We report a 58-year-old male with right lumbar pain radiating to the foot and intermittent paraesthesia. MRI revealed an extramedullary spinal mass compressing the right L5 nerve root and infiltrating the L5 vertebral body, with confirming the diagnosis of AML. PET-CT and follow-up MRI demonstrated extensive paraspinal and spinal canal infiltration. The patient initially developed rapid neurological decline with impaired gait and reflexes despite treatment, though subsequent combined systemic and intrathecal chemotherapy achieved partial regression in size and complete metabolic activity. The patient is currently planned for radiotherapy and stem cell transplantation. Spinal myeloid sarcomas are rare but clinically significant in patients with myeloid malignancies presenting with neurological deficits. MRI is the preferred imaging modality, but histopathology remains essential for diagnosis. Management generally requires systemic chemotherapy, with radiotherapy as adjunctive therapy. Prognosis remains guarded, particularly in cases complicated by spinal cord compression. Myeloid sarcoma should be considered in the differential diagnosis of spinal masses in AML. Early recognition and aggressive treatment resulted in a favourable outcome for our patient.

## Introduction

Myeloid sarcomas are defined as metastatic granulocytic solid tumours of myeloid origin at an extramedullary site [[1], [2], [3]]. The tumour was initially described by Burns in 1811, with King later naming it a “chloroma” due to the green colour which results from the myeloperoxidase in the immature cells [2,4]. The entity was renamed by Rappaport in 1966 owing to not all the cells having a green coloration, with various other colours resulting from different stages of cellular oxidation [2]. Nowadays, the term “myeloid sarcoma” is preferred by the World Health Organization.

Granulocytic sarcomas (GS) occur in up to 9.1% of patients with acute myeloid leukaemia (AML) but can also occur five times less frequently in patients with chronic myeloid leukaemia (CML) or myelodysplastic syndrome (MDS), with the overall prevalence being 2.9% when combining AML and CML [2,5,6]. Both sexes are affected in equal proportions, though children are more frequently affected with 60% of patients being younger than 15 years old [2]. However, the distribution appears bimodal, with an increase in the diagnosis of myeloid sarcomas in patients aged 20 to 44 years old [7]. Some genetic anomalies, such as a t(8;21) translocation have been shown to increase the risk of developing a myeloid sarcoma [4].

Granulocytic sarcomas can be clinically characterized in four groups: primary GS, GS at onset of acute AML, GS as an isolated recurrence of AML or as GS with a relapse of concurrent AML [8].

The most common sites for myeloid sarcomas to be found are soft tissues, connective tissues, breast tissue and the digestive tract, making up 50% of all cases [9]. The central nervous system is a rare site for a myeloid sarcoma to occur, with spinal myeloid sarcomas that result in nerve compression being even rarer [3]. The thoracolumbar spinal area is a rare location for a myeloid sarcoma to be found, with less than 100 cases reported in the literature to date [5,9].

Patients with AML will typically present with systemic symptoms, fatigue, weakness, petechiae, infections, cytopenia and weight loss, though they may also have neurological symptoms [10]. These nonspecific symptoms can make the diagnosis challenging [5]. However, patients can also have symptoms specifically related to the affected organs, which in the case of spinal cord compression can result in motor or sensory deficits or loss of bowel and bladder control [9].

We present the case of a 58-year-old man in whom a sacral and lumbar spinal myeloid sarcoma was discovered.

## Case report

A 58-year-old patient presented to the emergency department with right lumbar pain irradiating into his right foot with intermittent paresthesia. The patient described the pain as bearable when he was lying on his back, but the pain increased when standing. The patient also reported that walking had become increasingly difficult over the last 48 hours. The patient denied having a lack of control of bowel movements, urinary retention, or saddle anesthesia. Furthermore, he reported not having had any recent fever, nausea or vomiting. He had been self-medicating for pain with paracetamol, without relieving his symptoms.

The patient had a medical history of hypothyroidism but no relevant surgical or family history.

An MRI was ordered, revealing an extramedullary spinal mass that was compressing the right L5 nerve root, with invasion of the L5 lumbar vertebral body.

Bloodwork was then carried out, revealing normal hemoglobin (16.3 g/dl; *n* = 13.2 to 16.6). However, increasing undifferentiated blood cells were found (19.5%), with 1% consisting of myelocytes (*n* = 0%-0.1%) and 0.9% of erythroblasts. The patient also had mild thrombocytopenia (109,000 platelets/ml; *n* = 150,000 to 450,000).

Suspecting an oncological disease, the patient was transferred to our tertiary hospital to continue the diagnostic workup, where he was received three days later.

**Fig. 1:**
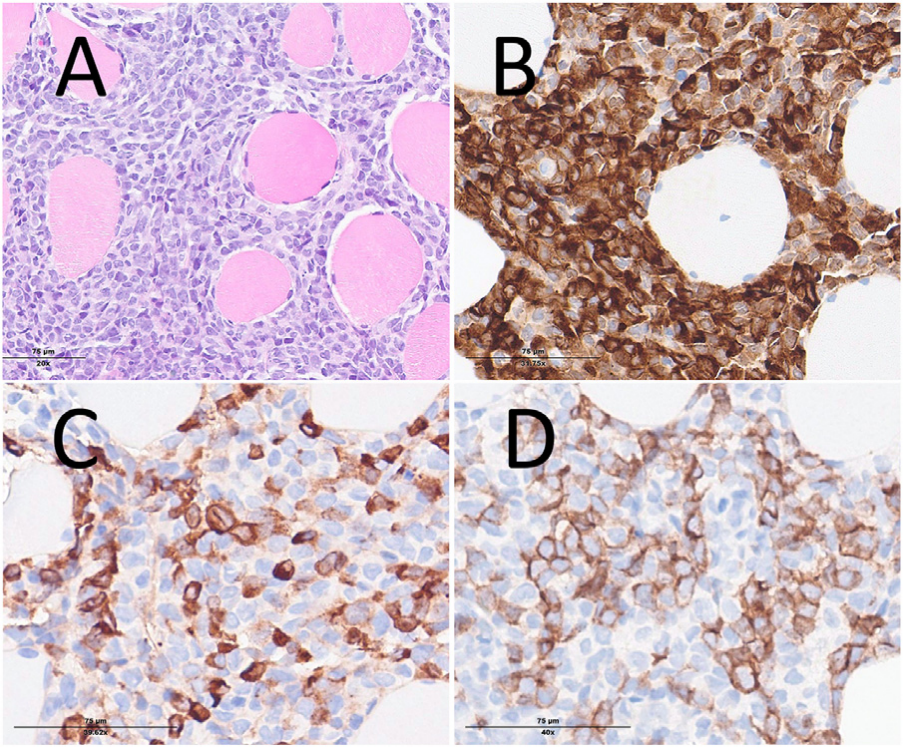
Histopathological image showing H&E staining of the bone marrow sample with 20X magnification (A), NPM1 markers with 31.75X magnification (B), MPO (myeloperoxidase) antibodies with 39.62X magnification (C) and CD177 markers with 40X magnification (D).

At the tertiary hospital, a bone marrow biopsy was performed, revealing >80% of myeloblasts. Another peripheral blood draw was carried out to perform blast immunophenotyping. This revealed that a minority of blasts were positive for CD34 (Cell surface glycoprotein), a marker for immature cells, but CD117 (c-Kit) expression was bright. Furthermore, HLA-DR (Major Histocompatibility Complex, Class II), CD33 (a marker for myeloid cells), CD13 (Aminopeptidase N), CD123 (IL-3 receptor α), CD4 (T-helper antigen), CD61 (Integrin β3), CD15 (Lewis X antigen) and CD65 (Carbohydrate antigen) markers were expressed. This led to the diagnosis of acute myeloid leukemia. Furthermore, molecular biology and next-generation sequencing demonstrated the presence of a mutated *NPM1 (Nucleophosmin 1)* and *FLT3-TKD (FMS-like Tyrosine Kinase 3 – Tyrosine Kinase Domain)* (Fig. 1).

The patient immediately started cytarabine and daunorubicin chemotherapy, based on the 7+3 protocol, and dexamethasone [11]. This was followed by the addition of midostaurine because of the *FLT3-TKD* mutation.

The patient’s condition continued to deteriorate, with the loss of his ability to walk two days later due to the pain. A neurological examination also revealed a loss of the patellar and ankle jerk reflex as well as an abnormal plantar reflex for his right leg, while the left leg remained unaffected. The patient also reported painful hypoesthesia and pain in the right L5 to S1 territory.

**Fig. 2:**
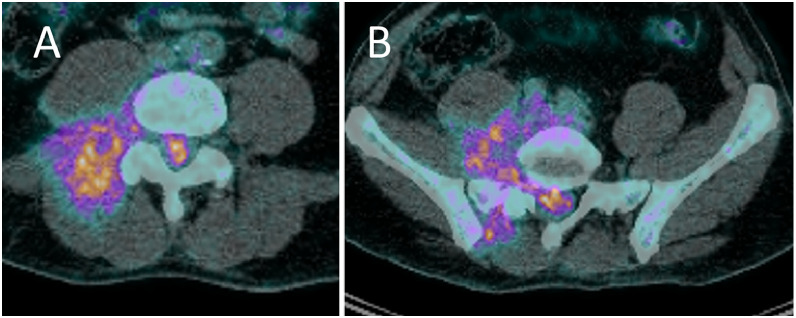
PET-CT image at the lower lumbar level (A) and upper sacral level (B) showing increased ^18^F-FDG uptake within the spinal canal, around the right L5 nerve root, around the right paravertebral muscle, as well as the right psoas and erector spinae muscles, corresponding to the location of the tumoural mass.

An ^18^F-FDG-PET-CT was ordered revealing an infiltrative mass around the right paravertebral muscle, as well as the right psoas and erector spinae muscles. The mass extended into the spinal canal and around the right L4 to S1 nerves (Fig. 2).

**Fig. 3:**
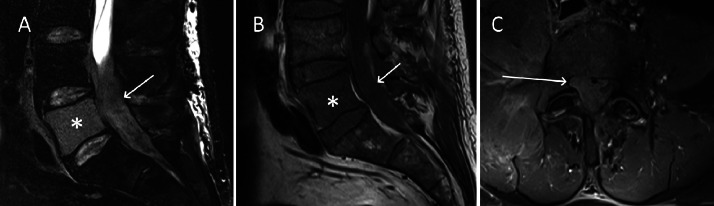
Sagittal T2-weighted image (A) showing a hyperintense L5 vertebra (star) corresponding to leukaemic tumoural infiltration of the bone and a heterogenously hyperintense epidural mass within the spinal canal corresponding to the tumour (arrow). Sagittal T1-weighted image (B) showing a hypointense L5 vertebra (star) when compared to the adjacent muscle, corresponding to tumoural infiltration of the bone. There is a hypointense epidural mass within the spinal canal, extending from L4 to S1 and into the right-sided L5-S1 foramen, corresponding to the tumour (arrow). Axial T1-weighted fat-saturated image acquired after gadolinium contrast administration showing an intensely enhancing mass within the spinal canal, corresponding to the myeloid sarcoma.

**Fig. 4:**
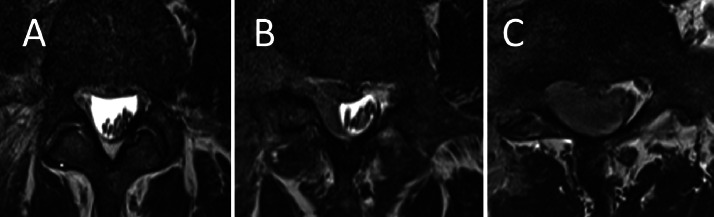
Axial T2-weighed images showing the progressive mass effect of the tumour within the spinal canal at the L4 (A), L5 (B) and S1 (C) levels, causing compression of the nerve roots.

The following a day, a second lumbar MRI (the patient’s first at our hospital) was performed to re-evaluate the extent of the mass around the nerve roots. This MRI showed an epidural mass within the spinal canal that was hyperintense in T2-weighted imaging (T2WI), isointense in T1-weighted imaging (T1WI) but greatly enhancing after contrast media administration, corresponding to a myeloid sarcoma (Fig. 3). This was accompanied by the complete replacement of the bone marrow signal of L5 by the tumor (Fig. 2). The mass within the spinal canal was compressing the nerves and infiltrating around exiting nerve roots on the right side (Fig. 4).

**Fig. 5:**
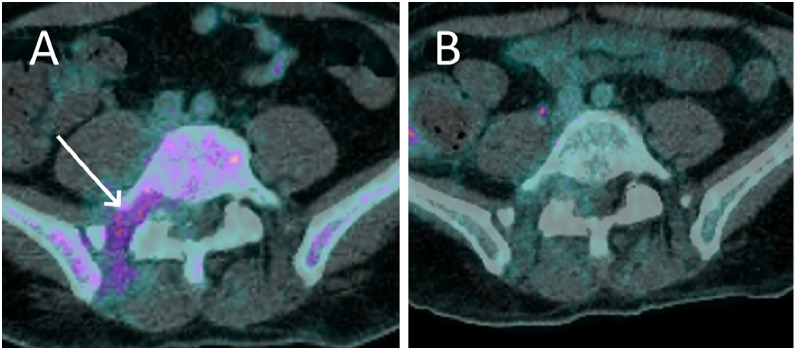
PET-CT image at S1 showing increased ^18^F-FDG uptake around the right paravertebral muscle and intervertebral foramen (A, arrow) corresponding to the location of the tumoural mass at one month after the initiation of treatment (A) and subsequent complete metabolic regression on the imagery performed two months after the initiation of treatment (B).

**Fig. 6:**
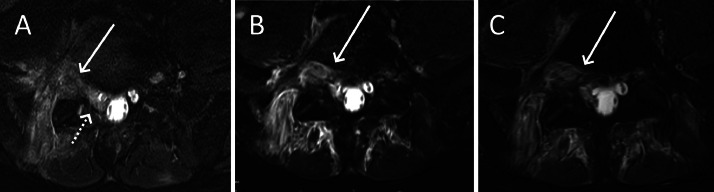
Axial T2 Dixon (water)-weighted image at the L5-S1 level showing the residual thickening around the right nerve root (solid arrow) during the 3rd MRI one month after the start of treatment with persistent intrathecal disease (dotted arrow) (A), the 4th MRI six weeks after the start of treatment (B) and the 5th MRI two months and a half after the start of treatment (C).

At one month after the patient first presented to our hospital, the patient was still undergoing chemotherapy. A follow-up PET-CT and MRI were performed at this time, as the patient has been given sufficient time to recover from the induction chemotherapy.The PET-CT showed marked regression of the hypermetabolism of the myeloid sarcoma (Fig. 5A) and the MRI showed a partial regression of the tumour, with significantly less compression of the dural sac (Fig. 6A).

Two weeks later (one month and two weeks after initial presentation at our hospital), the patient began the second induction chemotherapy. This was coupled with an intrathecal triple chemotherapy injection of cortisone, cytarabine and methotrexate on day 1, 4, 7 and 11. The spinal tap did not reveal any leukemic cells within the cerebrospinal fluid, neither in the microscopic evaluation nor by flow cytometry.

The patient then underwent a third MRI at two months after initial presentation at our hospital, revealing that the disease had stabilized but that the infiltration around the L5 nerve root persisted (Fig. 6B).

After day 11 of the second chemotherapy, intrathecal therapy was withheld due to severe thrombocytopenia following systemic chemotherapy. The patient also had a concomitant episode of agranulocytosis with subsequent uncomplicated diverticulitis, treated conservatively by piperacillin and tazobactam.

A third PET-CT and fourth MRI were performed three weeks later, corresponding to three months after initial presentation at our hospital, after recuperation from agranulocytosis after the second induction therapy. The PET-CT revealed a complete regression of the previous hypermetabolism of the myeloid sarcoma (Fig. 5B). The MRI showed a stable response with some residual thickening around the nerve root and the dural sac (Fig. 6C).

**Fig. 7:**
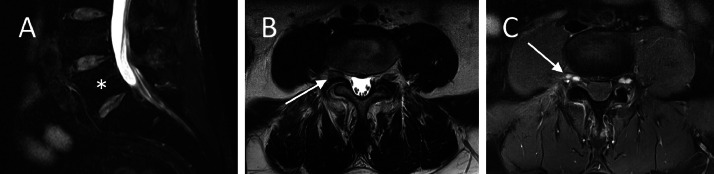
Sagittal T2-weighted image (A) showing a complete regression of the infiltration of L5 (star). Axial T2-weighted (B) and postcontrast T1-weighted fat-saturated sequence (C) image at the level of L5 showing complete regression of the infiltration of the right foramina and nerve roots (arrows).

The patient continues to receive 24 Gy of radiation therapy divided into 12 fractions of 2 Gy on the residual tumour on the right side of L5. In view of a stem cell transplant a pretreatment lumbar MRI was performed, showing complete regression of the disease (Fig. 7). The patient is currently awaiting an allogenic stem cell transplant.

## Discussion

Spinal myeloid sarcomas are rare extramedullary masses that generally occur in the context of AML and are composed of immature myeloid precursors [5,9].

As the tumours consist of myeloid precursors that are undergoing highly active proliferation, they are particularly sensitive to chemotherapy and radiotherapy, with many resolving within 3 months [2]. Recurrence is not uncommon, occurring in 23% of patients [2]. Paraspinal and intraspinal lesions are thought to occur due to dissemination of leukaemia cells into the arachnoid space [2]. This spread can result in neurological symptoms due to nerve compression [2].

The most common genetic mutation found to be associated with myeloid sarcomas in general is the *FLT3-TKD* mutation, being found in around 17.5% of patients [12]. To our knowledge, however, it remains unknown whether a specific mutation is associated with spinal myeloid sarcomas.

MRI is the diagnostic tool of choice for the diagnosis of spinal myeloid sarcomas [3]. Spinal myeloid sarcomas will generally appear T1 hypointense or isointense and T2 heterogeneously isointense or hyperintense to the adjacent white matter and enhance homogenously after gadolinium injection [1,2]. The intermediate signal in T2-weighted imaging can provide a valuable clue to when attempting to make a differential diagnosis between a myeloid sarcoma or a nerve sheath tumour, which will appear more hyperintense [13]. When imaged using CT it will appear as an isodense extra or intradural mass [2]. The most common site of spinal myeloid sarcoma is the epidural space, where it can compress the nerve roots [14]. Spinal myeloid sarcomas are most commonly found in the thoracic spine, followed by the lumbar, sacral and cervical levels, according to one study [14].

Axial bone myeloid sarcomas frequently occur, as was the case in our patient, who suffered vertebral body infiltration, due to the persistence of hematopoietic marrow [13]. These lesions can be lytic or sclerotic, though lytic lesions are more common [13,15]. Infiltration of the adjacent tissue is also common as the tumour spreads through the haversian canals, affecting ligaments and the periosteum [13,16]. MRI findings are nonspecific, with the lesions either being hypo- or isointense on T1-weighted sequences and slightly hyperintense on T2-weighted imaging [13]. There will, however, be marked enhancement after contrast is given [13]. When lesions are isointense in both T1 and T2-weighted imaging, this can help in differentiating the lesions from other bone tumours, osteomyelitis or lymphoma, which are hyperintense on T2-weighted imaging [13].

Other possible sites of dissemination include both extra- or intradural deposits within the skull, the mediastinum, the lungs, the pleura and pericardium, the hepatobiliary system, the urinary tract, the reproductive tract, the retroperitoneum, muscle, the skin and lymph nodes with heterogeneity of the radiological presentation being possible depending on the affected site [2,5,13].

Although imaging can suggest the diagnosis, a pathological analysis is required to confirm the diagnosis [2]. However, owing to the rarity of the disease, myeloid sarcomas are correctly diagnosed on imaging in around 50% of cases [3]. Another challenge can be myeloid sarcomas that occur without any hematological involvement, in which case they have been shown to proceed to bone marrow involvement within 10 to 11 months in 88% of patients [7].

The differential diagnosis included lymphomas, blastic plasmacytoid dendritic cell neoplasm, multiple myeloma, metastatic nerve sheath tumors and solid tumours such as carcinomas, as well as small blue cell tumours in children [4,13]. However, myeloid sarcomas should always be suspected when masses with unusual imaging features are discovered in patients suffering from acute or chronic myeloid leukemia and should not be mistaken for a secondary malignancy [13]. Clinical and pathological corroboration is therefore key to establishing the diagnosis [13].

Although there are no guidelines for the treatment of spinal myeloid sarcomas owing to the rarity of the pathology, the treatment can rarely involve surgical decompression if symptomatic [5,9]. However, surgery is only indicated in patients who deteriorate despite conservative treatment as there is no proven benefit for survival and it may delay chemotherapy [5]. Other treatment options include chemotherapy, radiotherapy, bone marrow transplants, steroids or a combination of the treatments [5,9]. The choice of treatment will depend on the individual characteristics of the patient and the biology of the leukemia, with radiotherapy being a possible adjunct when chemotherapy is insufficient [5]. Due to the paucity of evidence, the treatment recommendations rely on case reports and expert opinions [9].

The survival time is relatively short but ranges from 2.5 to 22 months, being worse in untreated patients, though complete remission is possible [5]. Furthermore, a recent literature review found that no case report exists wherein a patient suffering from a myeloid sarcoma with spinal cord compression has ever achieved remission, highlighting their poor prognosis [9].

## Conclusion

We present a case of 58-year-old man whose initial symptomatology of an AML was linked to a spinal myeloid sarcoma. The patient’s presentation with progressive neurological symptoms underscores the importance of including myeloid sarcomas in the differential diagnosis of spinal masses, despite this entity being rare. MRI is essential in diagnosing the condition, where it appears isointense or hypointense to the adjacent spinal cord and skeletal muscle in T1-weighted imaging and hyperintense in T2-weighted imaging, but enhances after contrast administration. Although one may suspect the diagnosis on imagery alone, pathology is required for the definitive diagnosis. Despite prompt initiation of chemotherapy and corticosteroids, the patient’s neurological status continued to decline, reflecting the aggressive nature of the disease and its poor prognosis, though subsequent treatment was able to lead to remission. Due to the rarity of spinal myeloid sarcomas and the lack of standardized treatment protocols, management is done on a case-by-case basis. Prompt diagnosis is essential to preserve neurological function and improve outcomes, although current evidence suggests that prognosis in such cases remains guarded.

## Patient consent

We hereby declare that we the patient provided informed consent that their data could be used for scientific purposes.
